# Glucose challenge test (50-g GCT) in detection of glucose metabolism disorders in peritoneal dialysis patients: preliminary study

**DOI:** 10.1007/s11255-014-0900-1

**Published:** 2014-12-25

**Authors:** Katarzyna Madziarska, Slawomir Zmonarski, Jozef Penar, Magdalena Krajewska, Oktawia Mazanowska, Hanna Augustyniak-Bartosik, Tomasz Gołebiowski, Renata Klak, Waclaw Weyde, Marian Klinger

**Affiliations:** 1 Department of Nephrology and Transplantation Medicine, Wroclaw Medical University, 213 Borowska St., 50-556 Wroclaw, Poland; 2 Faculty of Dentistry, Wroclaw Medical University, Wroclaw, Poland

**Keywords:** Peritoneal dialysis, Glucose challenge test, Glucose metabolism disorders

## Abstract

**Background:**

The aim was to evaluate the clinical utility of the oral glucose tolerance screening test (50-g GCT—glucose challenge test) for the detection of glucose metabolism disorders (GMD) in peritoneal dialysis (PD) patients with normal fasting glucose levels.

**Methods:**

The 50-g GCT was performed in 20 prevalent patients without history of diabetes before PD treatment onset, who had been on dialysis for a median time of 15.34 months. In addition, other indicators of glucose metabolism were measured: C-peptide, fasting insulin serum concentration, and the glycated hemoglobin level (HbA1c). The patients were prospectively followed for a median time of 25.8 months.

**Results:**

50-g GCT revealed GMD in 15 studied patients (75 %)—impaired glucose tolerance in 11 patients (55 %) and diabetes mellitus in four patients (20 %). HbA1c and insulin resistance, estimated by homeostasis model assessment, were elevated in two (10 %) and seven (35 %) patients, respectively. In patients with GMD, dietetic and pharmacologic interventions were performed. When the 50-g GCT was repeated at the end of the observation period, 12 (60 %) patients reported GMD, with no case of diabetes.

**Conclusion:**

50-g GCT appears to be a simple and practical tool for the detection of GMD in PD patients with normal fasting glucose. Timely therapeutic intervention can effectively inhibit the progression of glucose intolerance during PD treatment.

## Introduction

The pathogenesis of GMD in PD patients is related to factors such as weight gain and insulin resistance evoked by peritoneal glucose load [1–3]. Therefore, this group of patients should be mandatorily diagnosed for GMD. The periodical evaluation of PD patients for GMD is of clinical relevance and should be an element of the surveillance protocol. The “gold standard” method used for the diagnosis of GMD defined by the World Health Organization (WHO) in 1998 uses the level of 2-h post-challenge plasma glucose (2-hPG) in the oral glucose tolerance test (75-g OGTT) [4].

Criteria for the diagnosis are the following: fasting plasma glucose (FPG) 100 mg/dl (5.6 mmol/l) to 125 mg/dl (6.9 mmol/l) (impaired fasting glucose, IFG); 2-h PG in the 75-g OGTT 140 mg/dl (7.8 mmol/l) to 199 mg/dl (11.0 mmol/l) (impaired glucose tolerance, IGT); 2-h PG in the 75-g OGTT or random ≥200 mg/dl (11.1 mmol/l) (DM). The utility of the recently recommended use of HbA1c in terms of diabetes diagnosis (diagnostic threshold of ≥6.5 %) still remains to be validated [5]. The population at a particular risk of glucose metabolism disorders includes renal transplant recipients, pregnant women, and patients treated with peritoneal dialysis. For each of these groups, there is a lack of clear guidelines for the detection and diagnosis of GMD. Therefore, alternative methods for detecting all glucose metabolism disorders (IFG, IGT, and DM) in the population of high risk of hyperglycemia have been investigated.

The issue of glucose metabolism disorders (GMD) in PD patients has regained interest in recent years. This increase in clinical relevance was caused by new evidence that even mild hyperglycemia in the dialysis period can be harmful in the long term, being associated with worse survival and elevated risk of post-transplant diabetes mellitus [6–8].

Treatment with peritoneal dialysis is inherently associated with an additional load of glucose adsorbed from dialysis fluid. The effect of glucose deriving from the peritoneal cavity on the incidence of glucose intolerance still evokes some ambiguity, and published data are equivocal [1–3, 9–11]. The recent data suggest that high glucose exposure from dialysis solution may be a risk factor for vascular calcification in non-diabetic PD patients [12].

There seem to be some analogies between the occurrence of de novo GMD in patients treated with PD and gestational diabetes (GDM), in which a combination of increased maternal adiposity and naturally occurring human placental hormones produces insulin resistance [13, 14]. In both cases, the risk factors predispose to the onset of transient glucose intolerance. Gestational diabetes mellitus is defined as “any degree of glucose intolerance with onset or first recognition during pregnancy” [15]. The mechanisms that lead to chronic insulin resistance in GDM are varied as they are in the general population [13]. This distinction is emphasized even in diagnostic criteria which differ from the general population. Up to 2012 GDM was diagnosed by a two-step approach using a sequential model of universal screening with a 1-h 50-g glucose challenge test (50-g GCT) followed by a diagnostic 75-g oral glucose tolerance test (75-g OGTT) for women with a positive screening test [threshold value ≥140 mg/dl (7.8 mmol/l)]. The GDM was recognized in 75-g OGTT when the plasma glucose value was ≥140 mg/dl [16].

ADA recommendations for 2011 adopted new, stricter criteria for the diagnosis of gestational diabetes and confirmed them for 2012 [17]. According to the current recommendations for all pregnant women between 24 and 28 weeks of pregnancy, 75-OGTT should be performed. Gestational diabetes is diagnosed if the fasting plasma glucose is at least 92 mg/dl (5.1 mmol/l), after 1 h 180 mg/dl (10.0 mmol/l), and after 2 h 153 mg/dl (8.5 mmol/l). These criteria were based on the results of the HAPO study [18] and recommendations of the research group of the International Association of the Diabetes and Pregnancy Study Groups (IADPSG) [19].

There is also a critical discussion concerning the methods and criteria for the new ADA 2012 diagnosis of gestational diabetes [20–23]. Currently, there are no clear recommendations for diagnosis and classification of glucose disturbances occurring de novo in a population of patients on PD. In our opinion, the WHO “gold standard” is not appropriate for PD patients because GMD differ from those in the general population, and other screening strategies should be explored. Considering the mentioned similarities, we aimed to examine the utility of the oral glucose tolerance screening test (50-g GCT) for PD patients, which is used in a two-step approach to evaluate gestational diabetes. The first step—the 50-g GCT—is performed at any time of the day (the fasting state is not required), using 50 g of glucose, and the plasma glucose was measured at 1 h after administration.

In the current study, we aimed to evaluate the applicability of the 50-g GCT for the detection of GMD in non-diabetic continuous ambulatory PD patients with normal fasting glucose levels.

## Materials and methods

The study was performed in 20 prevalent patients without history of diabetes before PD treatment onset who had been on a PD program at our institution as their first dialysis modality. The median age of patients was 44.19 years [interquartile range (IQR) 34.15–56.25], 9 being males and 11 being females, all Caucasian with cause of end-stage renal disease (ESRD) being glomerular disease in 12 patients (60 %), hypertensive nephropathy in four patients (20 %), interstitial nephropathy in three patients (15 %), and polycystic kidney disease in one patient (5 %). The median duration of PD treatment before inclusion in the study was 15.34 months (IQR 10.8–21.34).

Patients were assessed at the study onset (on the day of the 50-g GCT) and at the end of PD treatment (13 patients) or at end of the observation period (December 2012) for continuing the PD program (seven patients). At the study onset, all patients underwent 50-g GCT with a glucose dose of 50 g. The test is performed at any time of the day (the fasting state is not required), using 50 g of glucose, and the glucose is measured at 1 h after the administration. Interpretation of our 50-g GCT results was as follows: glucose <140 mg/dl—normal value; 140–199 mg/dl—impaired glucose tolerance (IGT), ≥200 mg/dl—diabetes.

In case of a fasting glucose value 100–125 mg/dl, and normal value in 50-g GCT—impaired fasting glucose (IFG) was diagnosed.

The 50-g GCT was performed in patients with an empty abdomen after the night dwell draining. During the night, all patients used 1.36 % glucose containing dialysis solution.

The glucose was measured at 1 h after administration; the fasting state was not required. In addition, the following parameters of glucose metabolism were measured: C-peptide, fasting insulin serum concentration, and the glycated hemoglobin level (HbA1c). The concentrations of C-peptide and insulin were evaluated in serum samples, and HbA1c in whole blood using a chemiluminescent microparticle immunoassay (CMIA) on the ARCHITECT System (Abbott, USA). Based on fasting insulin and glucose values, insulin resistance (IR) was determined according to the homeostasis model assessment insulin resistance (HOMA-IR) [24].

Additionally, the available data from the patient surveillance charts were analyzed. They encompassed blood pressure (BP), serum albumin, lipid profiles, Hb, CRP, liver tests, mean daily peritoneal glucose load, ultrafiltration volume, residual diuresis, weekly dialytic creatinine clearance (CCr), weekly dialytic Kt/V, and other laboratory parameters (uric acid, PTH, TSH, Fe, TSAT, and electrolytes). Systolic and diastolic BP, residual GFR, weekly dialytic CCr, and Kt/V were measured by standard methods. The estimated peritoneal glucose load was calculated by the product of the volume and the glucose concentration of each exchange. The glucose load did not differ between the patients and was similar in all study patients during the follow-up. The patients were prospectively followed for a median time of 25.8 months (IQR 18.99–33.57), and 50-g GCT was repeated at the end of the observation period. The statistical analysis was performed with R for Windows, version 2.15.1, and MedCalc for Windows, version 12.3.1.0.

## Results

Clinical characteristics at the study onset are shown in Table 1.

**Table 1:** Clinical characteristics at the study onset

Variable	*N*	Median	IQR (interquartile range)
Fasting glucose level (mg/dl)	20	91.5	83.5–96
Glucose level—1 h after GCT (mg/dl)	20	165	137.5–195
HbA1c (%)	20	5.5	5.4–5.7
C-peptide (ng/ml)	20	7.24	5.12–10.95
Fasting insulin serum concentration (µU/ml)	20	10.34	7.79–16.05
HOMA-IR	20	2.13	1.57–3.53
Systolic BP (mmHg)	20	135	120–155.5
Diastolic BP (mmHg)	20	87.5	80–90
Serum albumin (g/dl)	20	3.8	3.65–3.95
Total cholesterol (mg/dl)	20	196	179–231
HDL cholesterol (mg/dl)	20	43	38–52
LDL cholesterol (mg/dl)	20	128.5	105.5–143
Triglycerides (mg/dl)	20	164.5	130–226.5
Hb (g/dl)	20	10.6	10.1–11.85
CRP (mg/l)	20	2.09	1.11–6.69
AspAT (IU/l)	20	17.5	14–21
AlAT (IU/l)	20	17	12–25
Uric acid (mg/dl)	20	6.3	5.55–7.3
PTH	20	817	490.5–1,221.5
TSH	20	2.19	1.05–4.11
Fe	20	74	51.5–88
TSAT (%)	20	25.4	19–33.9
Ca (mmol/l)	20	9	8.25–9.2
P (mg/dl)	20	6.4	4.85–7.25
Mean daily peritoneal glucose load (g/24 h)	20	120	120–120
Ultrafiltration volume (ml/24 h)	20	1,300	725–1,500
Residual diuresis (ml/24 h)	20	1,150	500–1,500
Weekly dialytic CCr	20	0.69	0.62–0.75
Weekly dialytic Kt/V	20	2.19	2.11–2.7
BMI—total (kg/m^2^)	20	25.14	23.83–27.63

50-g GCT revealed GMD in 15 investigated patients (75 %). The most frequent abnormality was IGT (plasma glucose between 140 and 199 mg/dl), which occurred in 11 patients (55 %). However, despite normal fasting glucose, in four patients (20 %), the glucose concentrations at 1 h after oral load were above 200 mg/dl, suggesting a preliminary diabetes diagnosis. The other glucose metabolism indicators exhibited little clinical utility for the detection of abnormal glucose tolerance in PD patients. The median percentage of HbA1c was in the normal range (5.5 %), exceeding the upper limit only in two patients (10 %). Median value of HOMA-IR was 2.13, being above 2.6 (the cutoff for IR) only in seven patients (35 %). C-peptide concentrations were significantly higher than in non-diabetic healthy individuals (*p* = 0.03). A significant correlation was found between HOMA-IR and C-peptide measurements (*p* = 0.001, *ρ* 0.677). HOMA-IR and C-peptide determinations did not correlate with other investigated parameters, i.e., serum albumin, CRP, lipid profile, glucose load in the dialysis fluid, dialysis dose, ultrafiltration, and residual diuresis.

There were no correlations between GCT results and uremic indices.

Patients with GMD were recommended a low-carbohydrate diet, lifestyle modification to promote weight loss, and increased physical activity. The adherence was checked at a monthly interval during patients’ visit to the PD clinic.

Four patients, in whom diabetes was recognized after the first 50-g GCT, additionally received short-acting sulphonylurea compounds (glipizide) for a few months (3–5), and then, a diet satisfactorily corrected the glucose value.

The patients were prospectively observed, and after a median time of 25.8 months, the glucose metabolism evaluation was repeated. During the second 50-g GCT, all patients were without antidiabetic medication. The GMD were confirmed in 12 patients (60 %). IGT persisted in eight patients (40 %), and of particular note, IFG (100–125 mg/dl) occurred in four patients (20 %) despite 50-g GCT being in the normal range. No diabetes case was recognized.

All patients who exhibited the highest abnormalities suggesting diabetes in the first GCT when retested at the end of the observation achieved a normal range.

Glucose metabolism disorders at study onset and at the end of observation are shown in Table 2.

**Table 2:** Glucose metabolism disorders at study onset and at the end of observation

GCT 50 g		
GMD	Study onset	End of observation
IFG	0 pts	4 pts
Fasting glucose level (mg/dl)		
Median/IQR		124/112–153
Glucose level—1 h after GCT (mg/dl)		
Median/IQR		176/152–189

IGT	11 pts	8 pts
Fasting glucose level (mg/dl)		
Median/IQR	93/83–95	87/78.25–90.25
Glucose level—1 h after GCT (mg/dl)		
Median/IQR	167/149–184	150/146–167

DM	4 pts	0 pts
Fasting glucose level (mg/dl)		
Median/IQR	93/83.3–99.8	
Glucose level—1 h after GCT (mg/dl)		
Median/IQ	207/204–236	

## Discussion

The occurrence of GMD in 75 % of the study subjects representing a small single-center cohort of prevalent PD patients gives a good illustration of the epidemiological dimension of the problem. After the first oral glucose load of 50 g, IGT was revealed in 11 subjects (55 %), and a glucose rise suggesting diabetes appeared in four patients (20 %). The 50-g GCT was performed in prevalent continuous ambulatory peritoneal dialysis (CAPD) patients, who had been on dialysis for a median time of 15.34 months.

Surprisingly, the literature on GMD in the PD population is very scanty. In two works published in the 1980s, Lindholm et al. [25, 26] based on their own modification of OGTT (using 1 g glucose per kg body weight) carried out in 15 patients concluded that PD treatment up to 1 year does not deteriorate glucose metabolism.

After a decade, Delarue et al. published results of OGTT diminished from standard 75–50 g glucose load accomplished in six patients being on PD for at least 6 months. The test was applied exclusively to detect insulin resistance, and the data on increased glycemic and insulinemic responses were presented [27]. More informative and extensive investigation was done by Cheng et al. [11] in 35 nondiabetic patients treated by PD for more than 1 year. They challenged patients by standard 75 OGTT and found IGT in 31 %.

In a larger-scale epidemiological study, insulin resistance was significantly more frequent in PD patients than in hemodialysis and pre-dialysis subjects (47 vs. 21 and 26 %, respectively) [28]. In a study by Tatar et al. [29], insulin resistance was an independent risk factor for arterial stiffness in nondiabetic PD patients older than 50 years.

In our present single-center study, we found HOMA-IR values indicating insulin resistance (>2.6) in seven patients (35 %).

The C-peptide levels were significantly higher in the PD group than in healthy individuals and correlated closely with HOMA-IR. It should be mentioned that the elevated C-peptide concentrations in PD patients are mainly a consequence of the fact that 70 % of this peptide is eliminated by the kidney route [30]. However, the strong correlation with HOMA-IR can be treated as a sign of preserved excretory *β* pancreatic cells capacity.

Commenting on the results of our study, it is worth emphasizing that the study group encompasses low-risk, middle-aged, non-obese subjects, with normal fasting glucose, normal HbA1c, and slightly elevated cholesterol level. The only more pronounced abnormality which is associated with glucose intolerance in epidemiological investigation was hypertriglyceridemia. Discovery of GMD in 75 % of patients with such clinical characteristics highlights the clinical significance of this issue.

We are conscious that 50-g GCT was not validated in the PD population, but it seems to be well suited for a person on PD treatment due to its simplicity and convenience. We performed only the first step—50-g GCT. In patients with a plasma glucose value ≥140 mg/dl, we gave up on performing the second step 75-g OGTT, because the aim of our study was not to recognize diabetes by WHO criteria, but reveal a group with special risk of hyperglycemia. We treated our test as a screening, not diagnostic method, also taking into account the cost–benefit relationship. Moreover, we did not want to expose patients to additional glucose loads.

The 50-g GCT allows quick verification of glucose disturbances in patients with an additional daily dose of glucose in the dialysis fluids. In contradistinction to standard 75-g OGTT, it does not require patients to keep coming to the PD clinic at different appointment hours without eating. It obviously does not provide a definitive diagnosis. In the next project following these preliminary data, we aim to compare the 50-g GCT with standard 75-g OGTT. However, we believe that abnormalities revealed by 50-g GCT are already of clinical value alerting the patient and physician. It provides a stimulus for preventive measures, which as shown by our experience can be effective, i.e., the normalization of the test in four patients with the worst results at the onset. The other positive aspect of the current study is supplying evidence that GMD are not inexorably progressive under PD treatment. The maintenance of normal glucose metabolism in PD patients is in the context of obtained results a feasible goal. Its achievement could bring benefits in terms of longer patient survival and lower risk of post-transplant diabetes development.

## Conclusion

The oral glucose challenge screening GCT test appears to be a sensitive instrument for the detection of glucose metabolism disorders in PD patients with normal fasting glucose.

Timely therapeutic intervention can effectively inhibit the progression of glucose intolerance during PD treatment.
